# Contralateral internal mammary vessels – a rescue recipient vessels option in breast reconstruction

**DOI:** 10.1080/23320885.2022.2048179

**Published:** 2022-03-10

**Authors:** Artur Nixon Martins, João Nunes Pombo, Catarina Paias Gouveia, Bruno Gomes Rosa, Gaizka Ribeiro, Carlos Pinheiro

**Affiliations:** Plastic, Reconstructive and Aesthetic Surgery, Centro Hospitalar Universitário Lisboa Norte, Lisbon, Portugal

**Keywords:** Breast reconstruction, DIEP, anastomosis, anastomosis conversion, free flap, case report

## Abstract

The most used vessels for free flap breast reconstruction are the internal mammary, the thoracodorsal and the circumflex scapular. We present a case where those were inadequate. DIEP vessels were passed through a created sternal groove and anastomosed to the contralateral IM vessels, accessed by the breast symmetrisation incisions.

## Introduction

The deep inferior epigastric perforator (DIEP) flap has become the gold standard for autologous breast reconstruction since its first report in 1994 [1–4]. When there is surplus abdominal tissue and no compromising regional surgery has been done, it supplies enough skin paddle and volume to recreate a natural breast. This is especially useful in radiotherapy-injured thoraces, since implant-based reconstruction has high risk of failure [5] and autologous techniques bring radiation-free tissues to the breast. Additionally, it allows to improve the abdominal contour in exchange for a new scar in an easily concealable location. Thus, it is a powerful body contouring reconstructive procedure with good level of patient satisfaction [6].

The thoracodorsal (TD) vessels were used as recipient vessels in the first description of this technique [1]. Even though both the axillary vessels and the internal mammary (IM) vessels can be used as recipient vessels, the latter are currently the preferred option for free flap breast reconstruction [7–14]. Recipient vessel conversion rate ranges from 2 to 20%, mainly due to recipient-donor vessels size mismatch and radiotherapy induced scaring or frailty [9,12,15].

Contralateral IM vessels have been used in bilateral breast reconstruction settings, unilateral breast reconstruction with contralateral autologous breast augmentation or bilateral breast augmentation [16–22]. All these authors planned to use the contralateral IM vessels preoperatively. We present a case of contralateral IM vessels as last resource recipient vessels for DIEP flap breast reconstruction, performing the anastomosis through the contralateral breast reduction symmetrization incision.

## Case report

**Figure 1.**
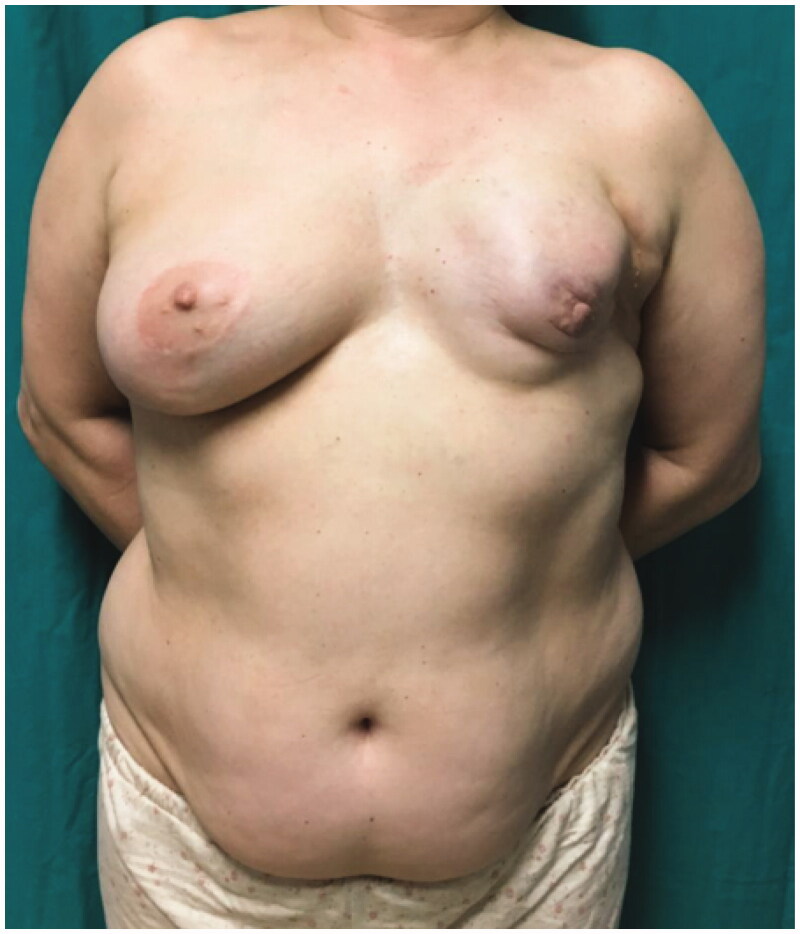
Preoperative photograph showing left breast deformity.

A 54-year-old woman, ex-smoker, presented with a dysmorphic left breast, after breast lumpectomy and radiotherapy, followed by implant-based reconstruction, 15 years ago (Figure 1). Due to the severity of capsular contracture and breast deformity, we opted for total breast reconstruction with DIEP flap and immediate symmetrization with vertical scar breast reduction.

**Figure 2.**
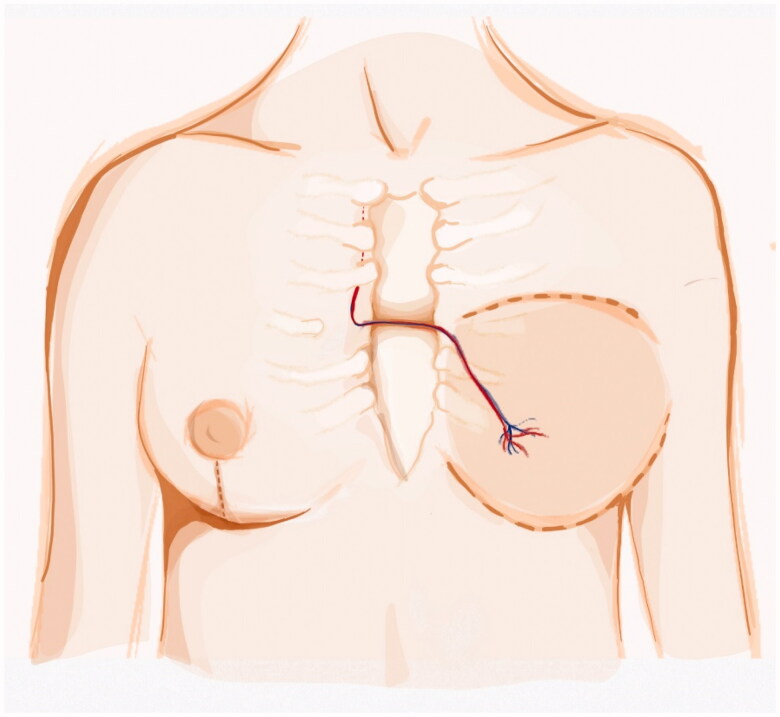
Left DIEP vessels anastomosed to the contralateral IM vessels. The pedicle is passed through an anterior sternal bony groove and the anastomosis were made possible by the symmestrization incision.

**Figure 3.**
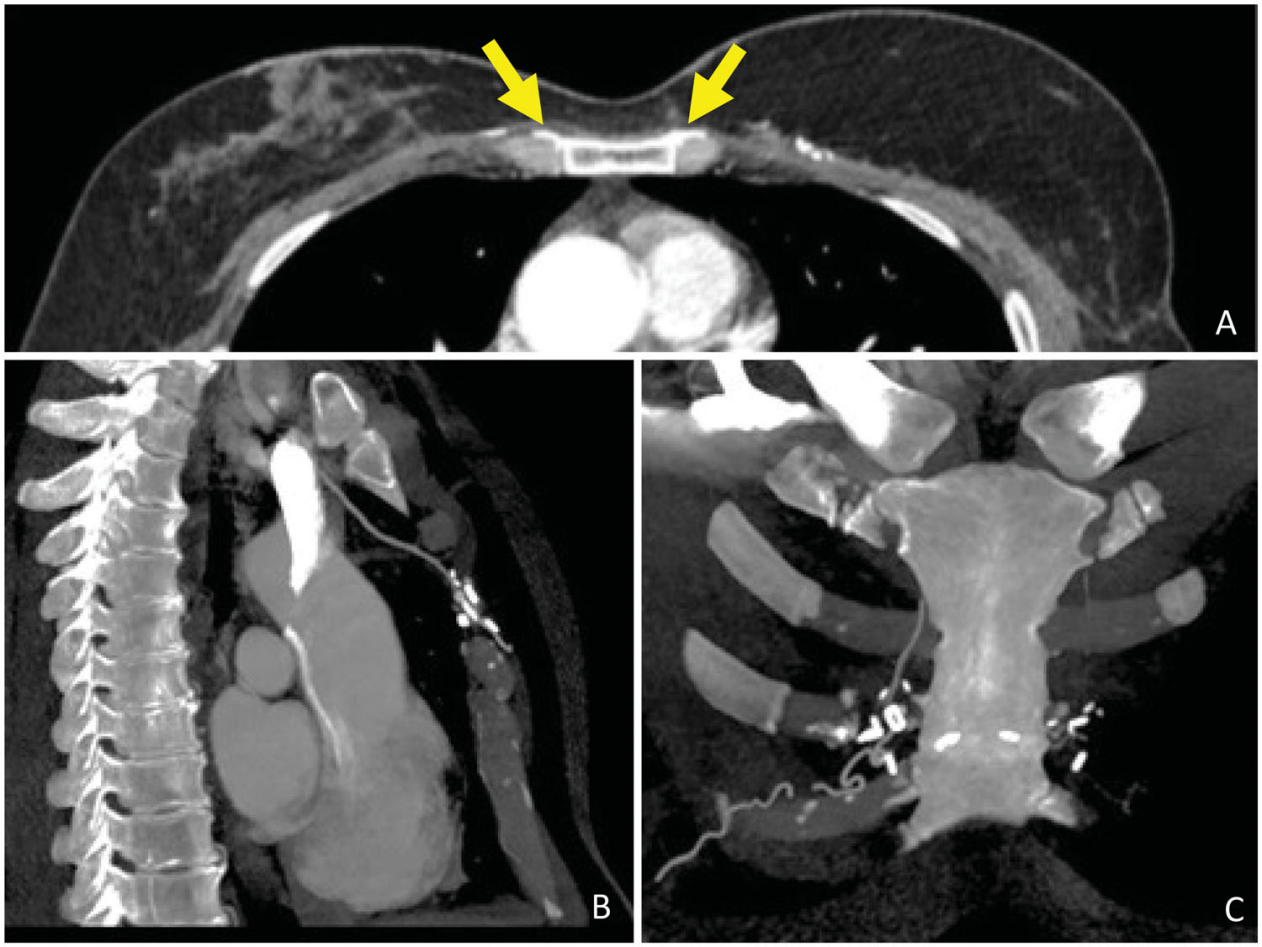
Postoperative CT scan. (A) Axial view, contrast enhancing shows DIEP vessels crossing the sternum (yellow arrows). (B) Sagittal view, IM vessels anastomosed to presternal vessels. (C) Coronal view, microvascular staples highlight the DIEP vessels path through the midline.

The ipsilateral IM artery was flowless and the IM vein had poor diameter. Both TD and circumflex scapular vessels diameters were negligible and with scarce blood flow. We opted for contralateral IM vessels as recipient vessels. To avoid vein collapse due to poor pliability of the presternal skin and sternal bone rigidity, a bony groove was scooped in the sternum to accommodate the pedicle. Anastomosis were performed through the symmetrization incision (Figure 2). A postoperative CT scan shows the pedicle crossing the sternum (Figure 3).

**Figure 4.**
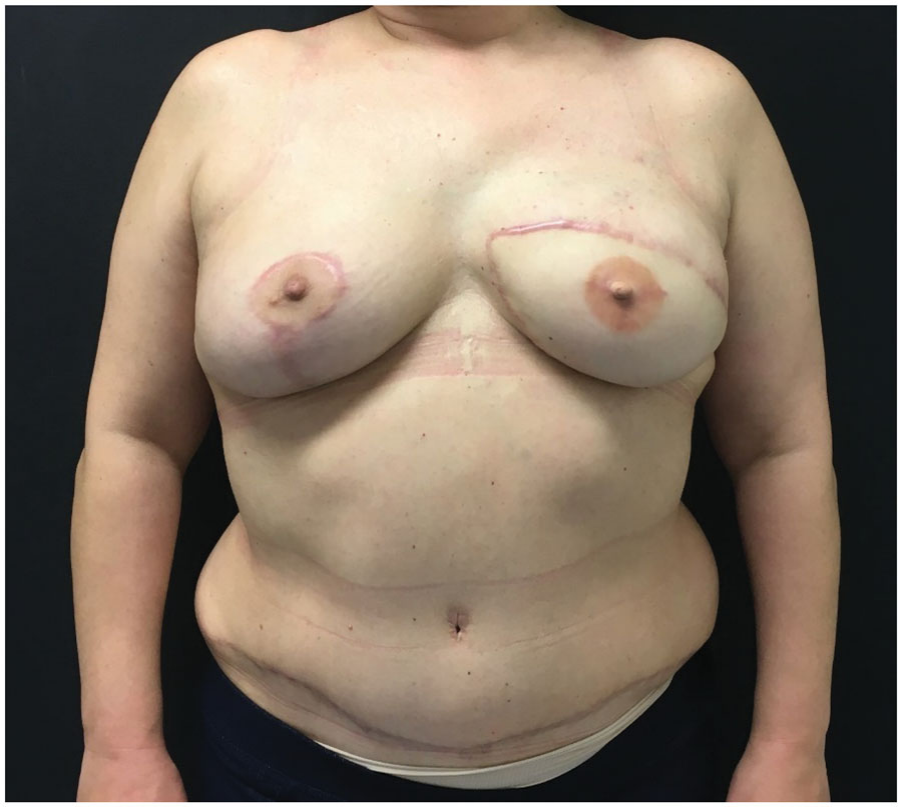
2-Year follow-up.

Despite midline thoracic pain during 6 weeks that were treated successfully with non-opioid analgesics the 2-year follow-up was uneventful (Figure 4).

## Discussion

The choice of recipient vessels is highly dependent on surgeon’s experience [8]. The current trend seems to favor IM, which our group support. It allows for a better diameter match with deep inferior epigastric vessels than TD [23]. IM artery blood flow rate is also higher than TD artery (mean 25 vs. 5 mL/min, respectively) [8]. Moreover, TD vessels usage precludes the *latissimus dorsi* flap as a salvage option in case of DIEP flap failure.

Recipient vessels unavailability is a limiting factor for microsurgery. Arteriovenous loops, venous grafts or usage of cephalic vein may be part of the solution. However, they increase scar burden. This may be a limiting factor in an aesthetically demanding reconstructive procedure. In free flap breast reconstruction the conversion recipient vessels options include the IM, TD, circumflex scapular, subscapular, serratus and lateral thoracic [9,23]. Other options include IM perforators [7] as well as distal end of IM vessels [22], however the flowless homolateral IM artery rendered these options unavailable. The need to conversion may be due to vessel mismatch, scarring, short pedicle, poor flow, vessel friability and small recipient vein or retrosternal location (in case of IM) [9]. Inadequate or absent IM veins is more commonly reported on the left side [9,24–26]. This relates with our case. Left side anastomosis is also more prone to venous thrombosis and overall complications [13,14].

There is conflicting data regarding radiotherapy and recipient vessel conversion rates [9,10,12,15]. However post-radiation sequelae include tissue fibrosis, edema, vasculitis, atherosclerosis and decreased IM artery diameter [14,27]. Intraoperative vascular complications seem to increase with radiotherapy [28]. In the present cast, IM artery insufficiency may be related to previous radiotherapy. Complications might be offset by postponing breast reconstruction for one year [14].

The literature so far presents anastomosis to contralateral IM vessel in cases where this option had been planned [16–21]. We did not plan to use these vessels preoperatively, but it became possible in view of the simultaneous symmetrisation procedure. Our team supports immediate contralateral balancing procedures as a way of enhancing overall breast satisfaction and reducing the chance of a second breast procedure [29–32]. This report further broadens the benefit of immediate breast balancing in free flap reconstruction in the rare cases of vessel insufficiency.

Other surgeons placed the pedicle in a subcutaneous plane [16–22]. There is no evidence that it leads to increased venous thrombosis rates. However, we theorize that the tight space between the presternal inelastic skin and the sternum would increase the tendency to venous collapse and thrombosis. Therefore, scooping a bony groove in the anterior sternal wall might reduce that risk. The drawbacks may be increased post-operative pain and increased surgical time.

Contralateral IM vessels might be a safe rescue recipient vessel option, especially in the immediate contralateral balancing setting, since it will not lead to an increased scar burden.
